# The Relationships of Microscopic Evolution to Resistivity Variation of a FIB-Deposited Platinum Interconnector

**DOI:** 10.3390/mi11060588

**Published:** 2020-06-12

**Authors:** Chaorong Zhong, Ruijuan Qi, Yonghui Zheng, Yan Cheng, Wenxiong Song, Rong Huang

**Affiliations:** 1 Key Laboratory of Polar Materials and Devices (MOE), Department of Electronics, East China Normal University, Shanghai 200241, China; 52161213009@stu.ecnu.edu.cn (C.Z.); rjqi@ee.ecnu.edu.cn (R.Q.); rhuang@ee.ecnu.edu.cn (R.H.); 2 School of Physical and Telecommunication Engineering, Yulin Normal University, Yulin 537000, China; 3 Erich Schmid Institute of Materials Science, Austrian Academy of Science, 8700 Leoben, Austria; Yonghui.Zheng@oeaw.ac.at; 4 State Key Laboratory of Functional Materials for Informatics, Shanghai Institute of Microsystem and Information Technology, Chinese Academy of Sciences, Shanghai 200050, China; 5 Collaborative Innovation Center of Extreme Optics, Shanxi University, Taiyuan 030006, China

**Keywords:** microscopic evolution, Pt interconnector, in situ heating and biasing, annealing treatment

## Abstract

Depositing platinum (Pt) interconnectors during the sample preparation process via a focused ion beam (FIB) system is an inescapable procedure for in situ transmission electron microscopy (TEM) investigations. To achieve good electrical contact and avoid irreversible damage in practical samples, the microscopic evolution mechanism of FIB-deposited Pt interconnectors need a more comprehensive understanding, though it is known that its resistivity could be affected by thermal annealing. In this work, an electron-beam FIB-deposited Pt interconnector was studied by advanced spherical aberration (Cs)-corrected TEM combined with an in situ heating and biasing system to clarify the relationship of microscopic evolution to resistivity variation. During the heating process, the Pt interconnector underwent crystallization, organic matter decomposition, Pt nanocrystal growth, grain connection, and conductive path formation, which are combined actions to cause several orders of magnitude of resistivity reduction. The comprehensive understanding of the microscopic evolution of FIB-deposited Pt material is beneficial, not only for optimizing the resistance performance of Pt as an interconnector, but also for understanding the role of C impurities with metal materials. For the purpose of wiring, annealed electron-beam (EB)-deposited Pt material can be recommended for use as an interconnector in devices for research purposes.

## 1. Introduction

With the development of transmission electron microscopy (TEM), there has been a growing interest in in situ TEM techniques to study the dynamic microstructure evolution of materials and devices induced by external heating/biasing/forcing/gas fields [1,2,3,4,5,6,7,8,9], which is characterized of high-spatial resolution and real-time recording. In situ TEM studies can clarify the structure–property relationships of advanced functional materials by recording their physical or chemical changes under external fields [10,11,12,13], which is helpful to optimize the performances of functional materials. For example, Gong et al. [8] fabricated an all-solid-state lithium-ion battery through a focused ion beam (FIB) system, and directly observed the in situ formation process of nano-polycrystals in TEM, which provides guidance for further optimizing battery properties. It is worth noting that the key point to guarantee the success of in situ TEM experiment is to fabricate a high-quality sample by FIB system, in which platinum (Pt) interconnector deposition [14,15,16] is an inescapable procedure. For the in situ biasing experiment the resistance of the Pt interconnector directly affects the electrical performance of device. Therefore, it is necessary to study the relationship between structure and property in Pt interconnectors for better applications.

In practice, a Pt interconnector can be deposited via the microchemical vapor-deposited method in a FIB system either by electron-beam (EB) or ion-beam (IB) deposition, in which the precursor is basically commercial cyclopentadienyl platinum-trimethyl [(CH_3_)_3_Pt(C_p_CH_3_)] [17,18]. Therefore, the obtained Pt interconnector is the mixture of Pt metal and organic compounds whose electrical properties will be affected by the structure of the mixture and organic dopant, i.e., carbon (C), resulting in high resistance. There are also some carbon-free precursors, such as tetrahedral [Pt(PF_3_)_4_] [19,20,21,22], which is expensive, is not desirable of the strong etching capability of F atoms, would decompose to produce poisonous PF_3_ gas [23], and so has not been utilized in large-scale commercial production. With commercial [(CH_3_)_3_Pt(C_p_CH_3_)] precursor, an IB-deposited Pt interconnector usually shows better electrical conductivity, which is suitable for connecting circuits [24]. More specifically, the resistivity of an IB-deposited Pt interconnector is around 70–700 μΩ·cm [25], while the value of the EB-deposited one is much higher, around 1–100 Ω·cm [26]. The difference between IB and EB deposition in resistivity is probably because the IB deposition process allows the organic precursor to decompose more completely than the EB method does [27,28]. However, the gallium (Ga) injection during the IB process will cause more irreversible damage [29,30,31,32], such as amorphization [33] or crystallization [34], which cannot be ignored. Therefore, the EB-deposited Pt interconnector [35,36] would be a compromise to have less damage but higher resistivity.

Recently, researchers found that the resistivity of the EB-deposited Pt material could be further reduced by means of heating treatment. For instance, its crystallinity could be improved when annealing under an N_2_ atmosphere, resulting in a reduced resistivity [37,38,39]. If the protective gas is replaced by O_2_, the resistivity of the Pt interconnector could drop by about three orders of magnitude, in which the deposition structure was purified due to the oxidation of carbonaceous material [36,39,40]. In terms of lowering the resistance of deposited Pt material, the annealing temperature increment plays a key role [41,42], while the annealing time has a slight effect [37,38,39]. Additionally, a freestanding Pt/C nanotip was skillfully deposited on a grid by IB to study the microstructure evolution, which was annealed to 500 °C and 900 °C, while the resistance of the nanotip was not obtained at the same time [43]. Though the structural change of Pt with temperature is probably the main origin of the resistivity reduction, a thorough explanation of the microscopic evolution with resistivity variation is still lacking, which is very important in understanding the underlying mechanism and further optimizing its resistance performance. In this work, in situ heating and biasing were carried out in a spherical aberration (Cs)-corrected TEM combined with the first-principles calculations to reveal the mechanism of resistivity variation from the microstructural aspect.

## 2. Experimental Methods

### 2.1. Materials and In Situ Experiments

**Figure 1:**
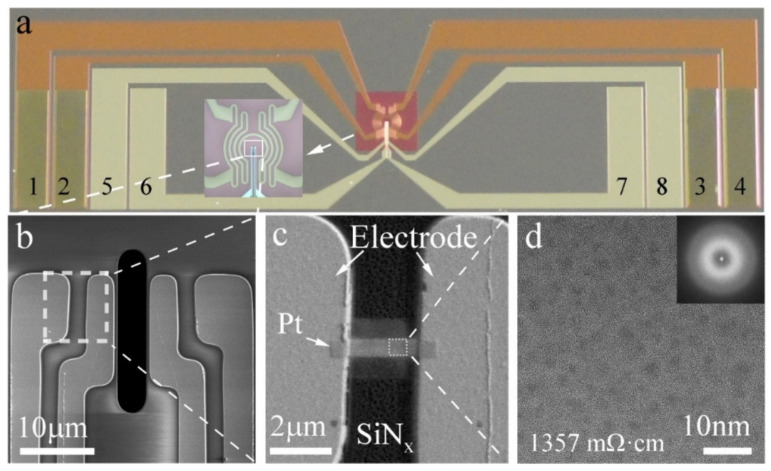
(**a**) Schematic of nano-chip for in situ heating and biasing together in transmission electron microscopy (TEM), in which, electrodes 1, 2, 3, and 4 are for heating, and electrodes 5, 6, 7, and 8 are for biasing. (**b**) Enlarged biasing electrodes. (**c**) Pt interconnector deposited between two electrodes on SiN membrane. (**d**) High resolution electron microscopy (HREM) image of the as-deposited Pt interconnector with inset of its corresponding Fast Fourier Transform (FFT) pattern revealing its amorphous structure.

A heating and biasing nano-chip from DENSsolutions (Delft, The Netherlands) with eight-point contacts was used for Pt interconnector deposition, as shown in Figure 1a. Considering the thickness of the SiN support membrane is relatively thick, etching from the back of the nano-chip to reduce its thickness is necessary to acquire an ultrathin SiN membrane between the two electrodes. Then, the Pt interconnector was deposited to connect two electrodes by using an electron beam (EB) in the FIB system (Helios G4, Thermo Fisher Scientific, Waltham, MA, USA). A gas injection system with cyclopentadienyl platinum-trimethyl [(CH_3_)_3_Pt(C_p_CH_3_)] in the FIB was used as the precursor for microchemical vapor-deposited. The size of the Pt interconnector is about 3 µm × 0.6 µm × 0.05 µm. EB deposition voltage: 2 kV; current: 1.6 nA; dose: 7.9 × 10^−10^ pC/um^2^; volume per dose: 5.0 × 10^−2^ µm^3^/nC.

The TEM characterization was carried out on JEM Grand ARM300F microscope (JEOL, Tokyo, Japan)operated at 300 kV with an image corrector. The composition analysis at different temperatures is obtained from the attached X-ray energy dispersive spectroscopy (JED-2200, JEOL, Tokyo, Japan). A Gatan Oneview camera was used for in situ recording HREM images with 25 frames per second. A Lightning D9+ heating and biasing holder from DENSsolutions (Delft, The Netherlands) was employed to manipulate the temperature and measure the resistance together. Temperature control was realized by Digiheater 3.2 software (DENSsolutions, Delft, The Netherlands). Resistance testing was done by using a Keithley 2450 external source measurement unit (Tektronix, Beaverton, OR, USA). The Pt interconnector is heated at a rate of 50 °C per minute and then held for about 5 min for thermal stability purposes and the related measurements. Compared to the irradiated experiment [44], the current density of the electron beam during the observation process was only ~0.02A/cm^2^, which can be ignored here.

### 2.2. First-Principle Calculations

First-principle calculations were carried out based on density functional theory (DFT). The Kohn–Sham equations were solved using the Vienna Ab Initio Simulation Package (VASP) [45,46,47,48]. The valence electron and core interactions were described using the projector augmented wave (PAW) method [49]. Electron exchange and correlation were described using the Perdew–Burke–Ernzerhof with van der Waals correction (PBE-D3) [50,51,52] generalized gradient approximation (GGA) [51] function with a kinetic energy cutoff of 520 eV, where the valence electrons treated are *5d^9^6s^1^* for Pt and *2s*^2^*2p*^2^ for C. The convergence criterion is 10^−7^ eV for electronic convergence and 0.02 eV/Å for force. A reasonably converged grid spacing of ~0.02 Å^−1^ is used.

## 3. Results and Discussion

### 3.1. Electrical Resistivity Results

The in situ heating and biasing experiment was conducted on the nano-chip, as shown in Figure 1a, in which electrodes 1, 2, 3, and 4 are for heating, and electrodes 5, 6, 7, and 8 are for biasing. This kind of surround heating type ensures the uniform temperature rise in the center biasing area [9]. Figure 1b shows the enlarged biasing area with four electrodes. The Pt interconnector was deposited between two of the electrodes on the SiN membrane after back-etching (in order to make the thickness suitable for TEM characterization) as shown in Figure 1c. In Figure 1d, the EB-deposited Pt interconnector manifests as an amorphous phase according to the homogeneous contrast in the high-resolution electron microscopy (HREM) image and diffuse rings in the inset fast Fourier transform (FFT) pattern. The resistivity of the as-deposited Pt interconnector was measured as 1357 mΩ·cm, which is too high to use as an interconnector.

### 3.2. Microstructure Evolution

**Figure 2:**
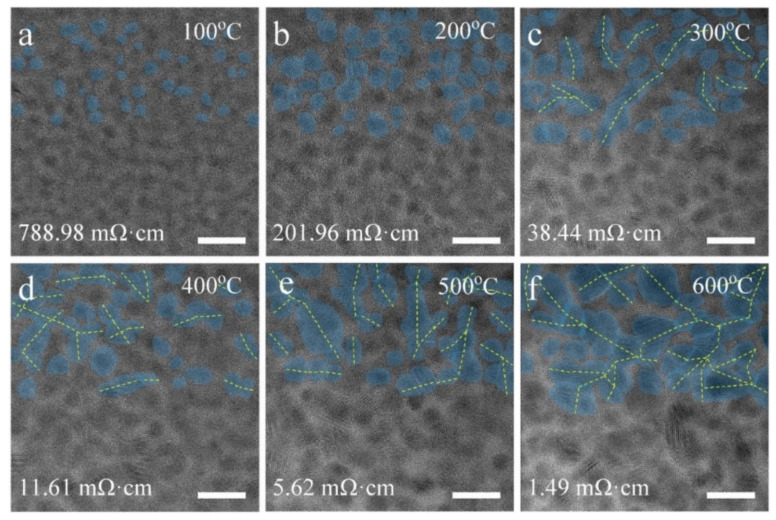
HREM images under different temperatures with their corresponding resistivity values of the Pt interconnector characterized by in situ heating and biasing in TEM together, revealing the gradual growth of Pt grains and the formation of the possible Pt conductive pathways. (**a**) 100 °C, (**b**) 200 °C, (**c**) 300 °C, (**d**) 400 °C, (**e**) 500 °C, (**f**) 600 °C. Scale bar = 10 nm.

**Figure 3:**
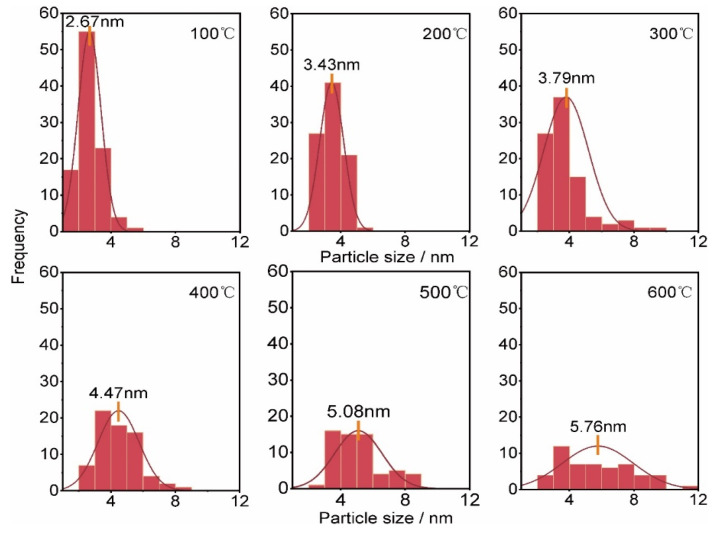
The distribution of the Pt particle size and corresponding fitted curves at different temperatures extracted from Figure 2, respectively. During the heating process, the number of particles gradually decreases and the average size increases due to the growth of nanograins.

To investigate the possible microstructure evolution with temperature of the EB-deposited Pt interconnector, an advanced in situ TEM technique was used to record a set of HREM images at different temperatures, as shown in Figure 2, in which the resistance is also measured simultaneously. When the Pt interconnector was heated to 100 °C, its resistivity was reduced from 1357 mΩ·cm to 788.98 mΩ·cm, as shown in Figure 2a, and small Pt nanocrystal grains began to appear in the film, in which some grains in the top half of the picture are painted blue. When the temperature increases to 200 °C, the grain size of the Pt nanocrystals becomes larger, and the number of Pt nanocrystals also increases, as shown in Figure 2b. At this temperature, the resistivity of the Pt interconnectors further decreases to 201.96 mΩ·cm. As the temperature continues to rise to 300 °C, these Pt grains grow further, and even merge to form the possible conducting pathways (denoted by dotted yellow lines), as shown in Figure 2c, accompanied with the continuous resistivity decreasing [43]. From 400 °C to 500 °C in Figure 2d,e, Pt resistivity continues to drop, while Pt grains further grow and combine, which benefits its conductivity. Increasing to 600 °C in Figure 2f, there is a significant increase in grain size, in which most of the grains are joined together to form a nanocrystal network. At this moment, the Pt resistivity has decreased by near three orders of magnitude compared with its initial state. Although its resistivity is still higher than pure bulk Pt metal (1.06 × 10^−2^ mΩ·cm), it can be used in in situ biasing research as an interconnector. The detailed Pt grain size distribution with temperature is also fully compared in Figure 3, which indeed shows that the density of nanograins gradually decreases and the average size increases during the in situ heating process.

### 3.3. Resistivity Transition Analysis

**Figure 4:**
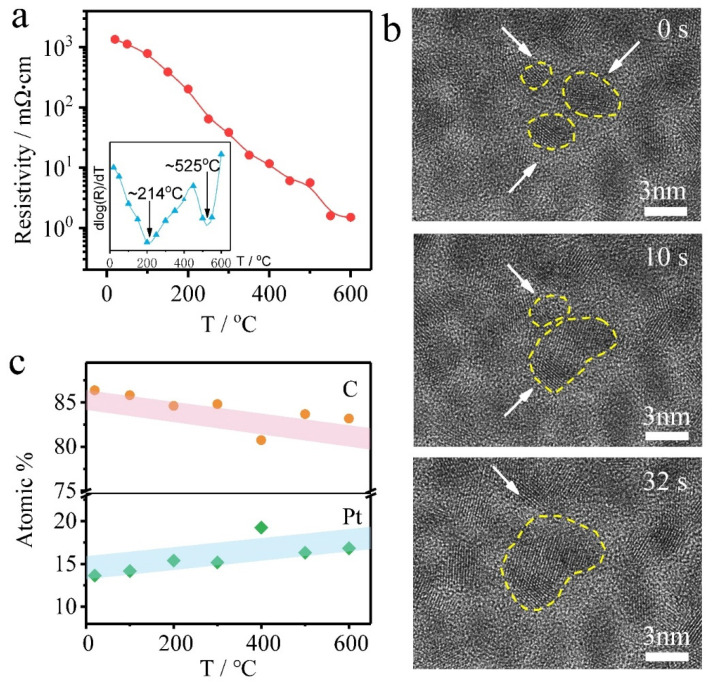
(**a**) Resistivity versus temperature curve of the Pt interconnector acquired from in situ biasing test in TEM and the inset is the transition temperature plots corresponding to the R-T curve calculated by dlog(R)/dT. (**b**) HREM images show the real-time grain growth and combination process during the in situ heating procedure at 250 °C. (**c**) The atomic percentage variation of C and Pt elements with temperature.

From the above results, it is clear that the crystallization, growth, and connection of Pt grains directly affect the electrical properties of the Pt interconnector. Hence, to further analyze the electrical property of the Pt interconnector, its resistivity as a function of temperature is plotted in Figure 4a, exhibiting a decreasing resistance trend. The inserted corresponding derivative of the ogarithmic resistivity to temperature (dlg*R*/d*T*) curve reveals two resistivity transition temperature at 214 °C and 525 °C, respectively. The first resistivity drop is due to the decomposition temperature of the organic Pt metal precursor material being around 250 °C [28], and also ascribed to the formation of conducting pathways, referring to Figure 2b,c. To obtain a better view, a real-time grain combination process lasting about half a minute at 250 °C is shown in Figure 4b, in which three small nanocrystal grains (denoted by yellow dotted lines) gradually merge to form a larger one (the detailed process can be seen in Supplementary Video S1). Additionally, the second resistivity drop, ascribed to the growth of Pt nanocrystals and the formation of a complete conductive network inside the Pt interconnector, is proven by the obvious grain growth from Figure 2e,f and Figure 3. Another concern is the possible composition variation of the Pt interconnector with increasing temperature. (CH_3_)_3_Pt(C_p_CH_3_) precursor includes elements Pt, C, and hydrogen (H). Considering that H is beyond the detection limit of energy-dispersive X-ray spectroscopy (EDS), we mainly concentrate on the composition variation between the C and Pt elements at different temperatures, as shown in Figure 4c. Conspicuously, the atomic amount of C decreases while Pt content increases with increasing temperature, indicating reducing organic content as reported in literature [35,37,39]. Therefore, the decrease of the organic matter content is also one of the reasons for the falling resistivity. On the other hand, the organic matter content still occupies the main component of the Pt interconnector, which is unfavorable to continue to improve its conductivity.

### 3.4. Lattice Constant of Pt Nanocrystal Evolution

**Figure 5:**
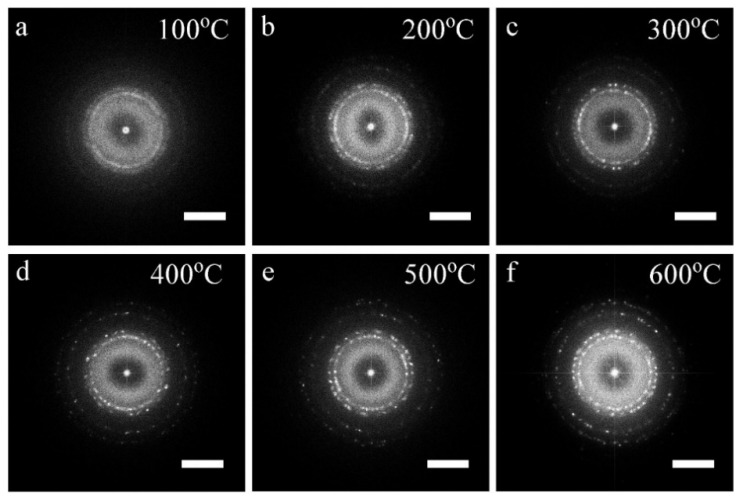
The Fast Fourier Transform (FFT) patterns extracted from HREM images in Figure 2. As the increasing of temperature, the polycrystal rings become more and more clear as shown in (**a**–**f**). At high temperature, some discrete spots can be found due to the growth of Pt nanograins. Scale bars, 5 1/nm.

**Figure 6:**
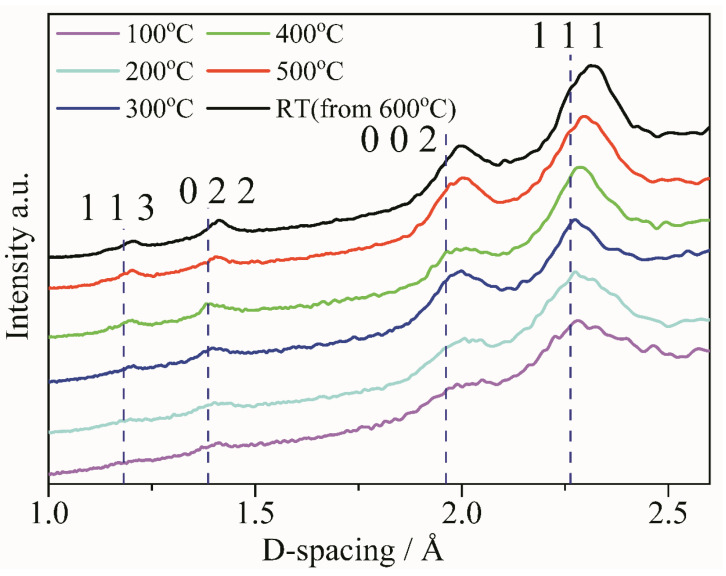
The structural evolution of the Pt interconnector with temperature in real-space obtained from the serial FFT patterns from HREM images at different temperatures.

Except for the structure, composition, and resistivity variation of the Pt interconnector during heat treatment, the lattice parameter may also change. To investigate the lattice parameter variation during the heating process, the FFT patterns at different temperatures were extracted from the HREM images (Figure 2), as shown in Figure 5. Afterwards, corresponding raw radially-integrated diffraction curves were obtained, as shown in Figure 6, in which the black dotted lines denote the position of the corresponding lattice planes in pure Pt ((JCPDS No. 04-0802). Inspecting the curves in Figure 6, the position of the peaks of (111) and (002) gradually shifts to the right side, and this phenomenon becomes more obvious above 400 °C, indicating the increasing d-spacings of the corresponding lattice planes of the Pt interconnector with temperature. The bias of the (022) and (113) peaks in Figure 6 are not so obvious, especially at lower temperatures, which may be due to the weakness of the corresponding rings and the poor signal-to-noise ratios and which are related to the crystallinity. After cooling to room temperature from 600 °C, the lattice parameter is still larger than that of the initial state, implying not merely a simple thermal expansion, but containing other factors.

### 3.5. The Probable Reason of Pt Lattice Parameter Variation

**Figure 7:**
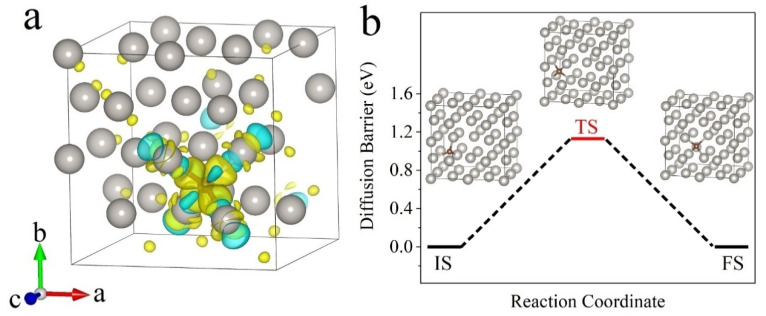
Calculation of the possibility of C atom doping into Pt lattice. (**a**) Charge density difference contours are plotted, in which, the yellow and cyan isosurfaces represent positive and negative charge density, respectively. (**b**) Calculated potential energy profiles between initial state (IS), transition state (TS), and final state (FS) when moving a tetrahedral C atom to another equilibrium tetrahedral position.

From the above results, the relationships of microscopic evolution to resistivity variation of the EB-deposited Pt interconnector are articulated clearly, and heat treatment can effectively reduce its resistivity. With the temperature increasing, Pt interconnector has undergone crystallization, organic matter decomposition, Pt nanocrystals growth, grain connection, conductive path formation, and lattice expansion, which are combined actions to cause a drop in resistance. Finally, the resistivity of the EB-deposited Pt interconnector still has a gap with pure Pt metal. Consider that, in addition to Pt metal, C is also an important component in this interconnector, which should be taken into account here. Since Figure 6 reveals the fact that Pt lattice is expanding and the characterization of C atoms is difficult in TEM, we attempt to investigate the possibility of C atom doping into the Pt structure via first-principle calculations. Figure 7 shows the calculation of one C atom in a tetrahedral position in the Pt lattice, which has a slightly lower formation energy than that in the octahedral position (Table 1). The positive formation energy demonstrates that C atoms do not desire to stay in the interstitial positions of Pt. After C doping, the distance of the second nearest neighbor Pt atoms is increased from 3.93 Å to 4.11 Å (Table 1), exhibiting lattice expansion consisting of the experimental result in Figure 6. Considering the electron transfer effect between C and Pt atoms, as shown in the charge density difference (CDD) map in Figure 7a, the formation of covalent bonds between C and Pt atoms localized the free electrons. Since the C atom obtains electron and its surrounding Pt atoms lose it, according to the Bader charge analysis, the electronic-carrier concentration would decrease, resulting in the conductivity of the Pt interconnector not reaching that of pure Pt metal [53,54]. However, the thermal stability of the interstitial C atom in the Pt lattice seems poor by calculating the diffusion energy barrier by moving a tetrahedral C atom to another equilibrium position, as shown in Figure 7b. The high diffusion barrier of 1.12 eV, as shown in Figure 7b, may result in a little C in the nterstitial positions from the kinetic perspective. Thus, the lattice expansion could be another reason; for instance, the grain size change of the Pt nanoparticles increased from the initial 2–3 nm to above 5 nm during the in situ heating process, which can also bring the change of the lattice parameter. Referring to the literature [55,56,57,58,59], there is a change in the opposite trend of the lattice parameter evolution to the particle size. For instance, no variation in the Pt-Pt distance was observed for Pt nanoparticles deposited on aluminum oxide films [57]. The unit cell parameter even linearly increases with the reducing particle size [59]. However, when introducing C into the Pt material system, Scardi et al. [55] found that the Pt lattice parameter would increase when the Pt particle is dispersed on the C support. A similar lattice parameter variation tendency is also confirmed for C-supported Pt nanoparticles, which is interpreted by the continuous-medium (CM) model [58,60]. Though C atoms are easily moved out of the Pt lattice, according to the calculation results, it may still be have an assistance effect to increase the Pt lattice parameter when the nanoparticles grow. Thus, C still tends to stay outside the Pt nanocrystals and form composites with Pt, which is the reason that the conductivity of annealed the EB-deposited Pt interconnector is not as good as that of pure Pt metal:

*E*_f_ = *E*_total_ − *E*_Pt_ − *E*_C_, (1)

where *E*_total_, *E*_Pt_, and *E*_C_ are the energy of the system, pure Pt, and one C atom, respectively.

## 4. Conclusions

An EB-deposited Pt interconnector by FIB is an appropriate choice for in situ TEM studies, while the relatively higher resistivity can be modified. In this report, with the help of in situ heating and biasing in TEM together, the crystallization of the EB-deposited Pt interconnector with temperature has been observed, while the combination of crystal Pt grains could form conductive pathways, even networks, resulting in an effective resistivity reduction. Combined with the above reasons, three orders of magnitude of resistance decreasing after annealing to 600 °C makes the EB-deposited Pt interconnector suitable for the electrical circuit. These results can help to understand the structure–property relationships of the Pt interconnector deposited by a FIB system, and to optimize the research scheme in designing reasonable in situ experiments more efficiently.

## Figures and Tables

**Table 1:** The formation of energy and structural features after doping one C atom in tetrahedral or octahedral interstices in Pt lattice, in which, the formation energy was calculated using the formula,.

Structure	*E*_f_ (eV)	^III^*d*_1_ (Å)	^IV^*d*_2_ (Å)	^V^*q* (e^−^)
pure	0	2.77	3.93	0
^I^ tetrahedron	2.07	3.13	-	0.57
^II^ octahedron	2.14	-	4.11	0.82

I is one C atom in tetrahedral interstice; II is one C atom in octahedral interstice; III is the distance of the first nearest neighbor; IV is the distance of the second nearest neighbor; V is the Bader charge for C. Inspecting the formation energy of interstitial C atom in different positions, the tetrahedral position is 2.07 eV, which is slightly lower than that in the octahedral position with 2.14 eV.

## Supplementary Materials

The following are available online at https://www.mdpi.com/2072-666X/11/6/588/s1, Video S1: The real-time grain growth and combination process.
